# Dual gene transfer of bFGF and PDGF in a single plasmid for the treatment of myocardial infarction

**DOI:** 10.3892/etm.2014.1485

**Published:** 2014-01-14

**Authors:** KAIJUN CUI, XIKUN ZHOU, JINGWEN LUO, JIAYUE FENG, MINGXIA ZHENG, DEJIA HUANG, JIAN JIANG, XIAOPING CHEN, YUQUAN WEI, JIONG LI, LI YANG

**Affiliations:** 1 Department of Cardiovascular Medicine, West China Hospital, Sichuan University, Sichuan 610041, P.R. China; 2 State Key Laboratory of Biotherapy, West China Hospital, Sichuan University, Sichuan 610041, P.R. China; 3 Institute of Parasitic Disease Control and Prevention, Sichuan Center for Disease Control and Prevention, Chengdu, Sichuan 610041, P.R. China

**Keywords:** acute myocardial infarction, basic fibroblast growth factor, platelet-derived growth factor

## Abstract

Basic fibroblast growth factor (bFGF) and platelet-derived growth factor (PDGF) have been shown to be involved in a spectrum of cellular processes. In a previous study, we constructed a novel multigenic vector that contained two separate transcription units, each consisting of a strong promoter and an efficient polyadenylation signal. The two promoters were chosen for their ability to work simultaneously. Dual gene transfer of bFGF and PDGF in a single plasmid resulted in a significant increase in collateral blood vessel formation in a rabbit model of hind limb ischemia. The aim of the present study was to investigate the effect of this dual gene transfer strategy in a rat model of acute myocardial infarction (AMI). AMI was induced in rats by ligation of the left anterior descending coronary artery. The animals were randomly divided into four groups and treated with the following therapeutic strategies: Empty plasmid (control), plasmid encoding bFGF (PL-bFGF), plasmid encoding PDGF (PL-PDGF) or plasmid encoding bFGF and PDGF (PL-F-P). Echocardiography and histological examinations were performed 28 days subsequent to gene transfer. Dual gene therapy with bFGF and PDGF resulted in a significant angiogenic effect accompanied by vessel maturation, along with a significant reduction in infarct size and improvement in cardiac function. In a rat model of AMI, single plasmid-mediated dual gene therapy with bFGF and PDGF decreased infarct size and improved cardiac function due to the formation of functionally and morphologically mature vasculature. These results are relevant to the ongoing clinical trials involving the use of single plasmid-mediated angiogenic factors for the treatment of myocardial ischemic disease.

## Introduction

Mechanical revascularization during percutaneous coronary intervention (PCI) and coronary artery bypass grafting (CABG) is largely effective in treating patients with myocardial infarction; however, there are certain categories of patients in which mechanical revascularization techniques are not able to be applied, and this is affecting an increasing number of patients. This is particularly true for patients with extensive ischemic tissue and patients who have undergone multiple bypass and stenting procedures (1,2). The new strategy of therapeutic angiogenesis was developed for the treatment of such patients.

A number of growth factors have been demonstrated to induce angiogenesis. Basic fibroblast growth factor (bFGF) is a potent mitogen for cells of mesenchymal, neural and epithelial origin, including all the cell types found in the vascular wall (endothelial cells, smooth muscle cells and pericytes) (3). Preclinical studies have demonstrated that the application of bFGF may lead to the development of collateral circulation, accompanied by the restoration of myocardial perfusion and an improvement in cardiac function (4,5). The application of bFGF has been shown to be safe and potentially efficacious for the treatment of ischemic disease in a number of small clinical trials, including a phase I randomized, double-blind, placebo-controlled trial (6–8). Platelet-derived growth factor (PDGF), which recruits smooth muscle cells and pericytes to promote extracellular matrix deposition and stabilize neonatal vessels, has been demonstrated to induce the formation of larger, more mature vessels (9–12).

In our previous study, a novel multigenic vector with two transcription units, allowing the combined expression of two genes of interest from a single vector, was constructed (13). In the study, the synergistic effects of bFGF and PDGF were examined on therapeutic angiogenesis in a rabbit model of hindlimb ischemia, using an intramuscular injection of naked plasmid DNA. It was observed that the transient combined delivery of bFGF and PDGF naked DNA resulted in greater increases in capillary growth, collateral formation and popliteal blood flow compared with those following the control and single gene delivery (13). In the present study, the therapeutic effect of this multigenic vector encoding bFGF-2 and PDGF was examined in a rat model of acute myocardial infarction (AMI), with the aim of verifying whether this multigenic vector-based dual gene delivery of bFGF-2 and PDGF has a potential application in the treatment of myocardial ischemic disease.

## Materials and methods

### Animal model

Male Sprague-Dawley rats (weight, 200–250 g) were obtained from the Laboratory Animal Center of Sichuan University (Chengdu, China). All animals were handled in strict accordance with good animal practice, as defined by the relevant national and/or local animal welfare bodies, and in accordance with the recommendations in the Guide for the Care and Use of Laboratory Animals of the National Institutes of Health (8th edition, 2011). The study was approved by the Ethics Review Board for Animal Experiments of Sichuan University.

### Construction of recombinant plasmids

**Figure 1:**
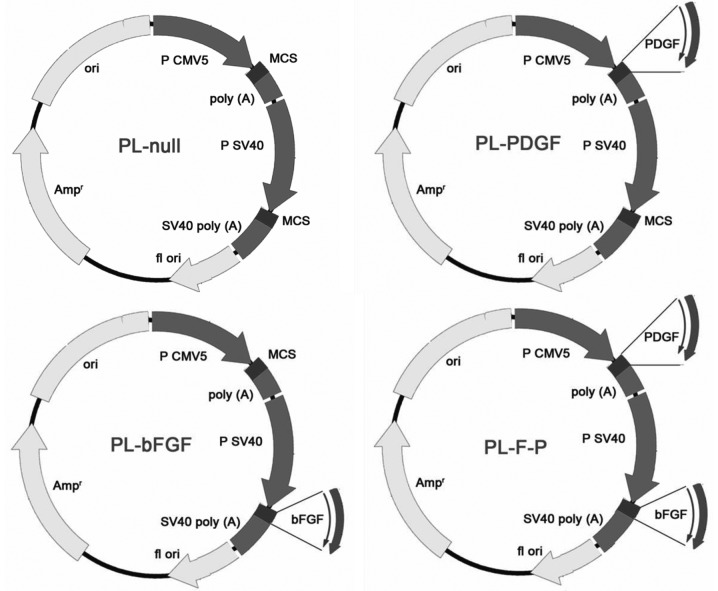
Schematic diagram of the plasmids encoding basic fibroblast growth factor (PL-bFGF), platelet-derived growth factor (PL-PDGF), and bFGF and PDGF (PL-F-P). Two transcription units allowing the combined expression of two genes were first assembled into a single vector. bFGF and PDGF-BB (PDGF homodimer) genes were amplified by polymerase chain reaction (PCR). The amplified fragment was digested with restrictive enzyme and inserted into the multigenic vector. SV40, Simian virus 40. MCS, multiple cloning site.

A CMV5 promoter/enhancer and β-globin poly(A) region from a pAdenovator-CMV5 vector were inserted upstream of the Simian virus 40 (SV40) enhancer and early promoter sequence in the pSI expression vector using multi-step polymerase chain reactions (PCRs). The bFGF and PDGF genes were amplified from pBLAST45-hbFGF and pBLAST49-hPDGF, respectively, and then cloned into the newly constructed double-promoter plasmid (Fig. 1; Takara Tech., Dalian, China). The resulting construct was identified using PCR, restriction endonuclease digestion and sequence analysis.

### Rat models of AMI and treatment

AMI was induced in the anterolateral wall of the left ventricle by ligation of the left anterior descending coronary artery. The rats were randomly assigned to one of four groups (n=10 rats per group): i) Rats treated with control plasmid (PL-Null); ii) rats treated with plasmid encoding bFGF (PL-bFGF); iii) rats treated with plasmid encoding PDGF (PL-PDGF); iv) rats treated with plasmid encoding bFGF and PDGF (PL-F-P). The PL-Null group was referred to as the control. Ten minutes subsequent to AMI, the border zone of the infarct area was directly injected with plasmid (100 μg in 100 μl) between the infarcted and normal tissue using a 1-ml syringe and a 25-gauge needle. All the rats were closely monitored and were treated with antibiotics.

### Functional echocardiography assessment

Echocardiography was performed in all the experimental subjects 28 days subsequent to gene transfer, under controlled anesthesia and using a 3–8 MHz phased-array transducer (Olympus Corporation, Tokyo, Japan) and Philips Sonos 7500 ultrasound system (Philips Healthcare, Andover, MA, USA). M-mode tracing and 2D echocardiography images were recorded from parasternal long- and short-axis views. The short-axis view was at the level of the papillary muscles. Left ventricular (LV) end-systolic and end-diastolic dimensions, as well as systolic and diastolic wall thickness were measured from the M-mode tracings. For each M-mode measurement, ≥3 consecutive cardiac cycles were sampled. The average LV end-diastolic diameter (LVDd), LV end-systolic diameter (LVDs), LV ejection fraction (LVEF) and LV fractional shortening (FS) were measured. All measurements were performed by two experienced echocardiographers who were blinded to the treatment group.

### Assessment of fibrosis using Masson’s trichrome staining

The rats were sacrificed 28 days subsequent to gene transfer. The hearts from each group were fixed in 4% paraformaldehyde, embedded in paraffin, sectioned transversely and stained with Masson’s trichrome. Sections from each heart were evaluated in their entirety and quantified using a computer-assisted automated image analyzer (Image ProPlus; Media Cybernetics, Inc., Rockville, MD, USA). The extent of fibrosis was assessed by measuring the collagen area as a proportion of the total LV area. Morphometric studies were performed by two examiners blinded to the treatment group.

### Blood vessel measurements

The rats were sacrificed 28 days subsequent to gene transfer. Heart muscle samples were fixed in 4% paraformaldehyde, embedded in paraffin and sectioned transversely. Endothelial cells were identified by the expression of von Willebrand factor (vWF), while smooth muscle cells were identified by the expression of smooth muscle actin (SMA). Immunohistochemistry was performed with specific primary antibodies against vWF (1:50; Dako Denmark A/S, Glostrup, Denmark) or SMA (1:2,000; Sigma, St. Louis, MO, USA). Vessels from the infarction border zone were analyzed at ×200 and ×400 magnification for SMA and vWF immunostaining, respectively. All vessel measurements were performed in a blinded manner in six randomly selected fields on the infarction border zone from each section. Blood vessel density was expressed as vessel number/mm^2^.

### Statistical analysis

Data are expressed as the mean ± standard error of the mean (SEM) and were analyzed using SPSS version 11.0 statistical software (SPSS, Inc., Chicago, IL, USA). Differences between groups were analyzed using analysis of variance (ANOVA) and the least significant difference (LSD) method. P<0.05 was considered to indicate a statistically significant difference.

## Results

### Changes in cardiac function following gene transfer

Twenty-eight days subsequent to infarction, the PL-F-P group had a significantly lower LVDd, and LVDs than the PL-bFGF and PL-PDGF groups (P<0.01). Each of these three groups had a significantly lower LVDd, and LVDs than the control group (P<0.01). Conversely, the PL-F-P group showed a significantly higher level of LVEF and FS than that of the control group (P<0.01; Table I). These results suggest that improved cardiac function was obtained following the dual gene transfer of bFGF and PDGF.

### PL-F-P injection reduces fibrosis of the infarcted myocardium

**Figure 2:**
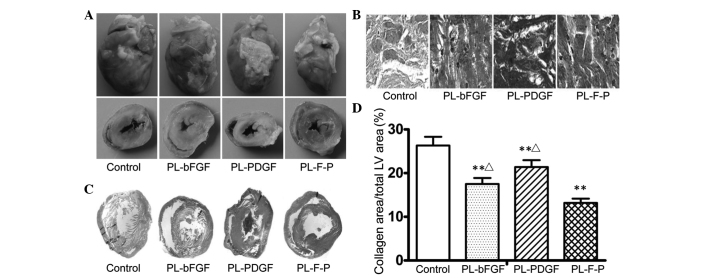
Injection with plasmid encoding bFGF and PDGF (PL-F-P) reduces fibrosis of the infarcted myocardium. (A) The hearts of the rats in each group in general and cross-section. (B) Representative samples of myocardial section stained with Masson’s trichrome (magnification, ×10). (C) Representative samples of myocardial section stained with Masson’s trichrome (magnification, ×400). (D) Quantitative analysis revealed that the average infarct size in all the therapy groups was smaller than that in the PL-Null treated group at 28 days subsequent to infarction (P<0.01). Among the three recombinant plasmids, PL-F-P showed the most marked effect on infarct area. ^**^P<0.01 versus the control group; ^△^P<0.05 versus the PL-F-P group. bFGF, basic fibroblast growth factor; PDGF, platelet-derived growth factor; PL-bFGF, plasmid encoding bFGF; PL-PDGF, plasmid encoding PDGF; LV, left ventricular.

Histological examination revealed that the average infarct size in all the therapy groups was smaller than that in the PL-Null group at 28 days subsequent to infarction (Fig. 2). Compared with the infarct size in the PL-Null group, the average infarct size in the PL-bFGF, PL-PDGF and PL-F-P groups was reduced by 30.0, 18.0 and 46.7%, respectively. PL-F-P showed the most marked effect on infarct size. The average infarct size in the PL-F-P group was 23.7% smaller than that in the PL-bFGF group and 33.4% smaller than that in the PL-PDGF group.

### PL-F-P injection increases vascular density

**Figure 3:**
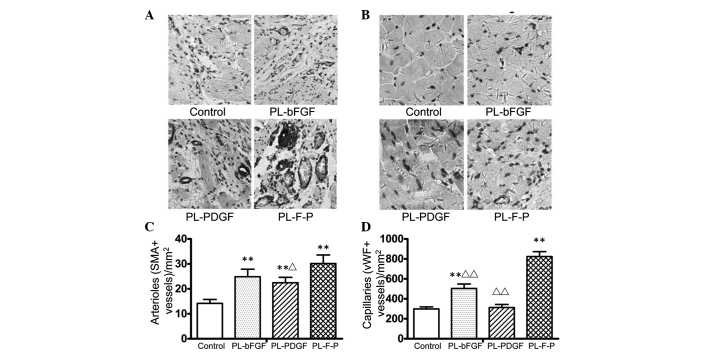
Injection with plasmid encoding bFGF and PDGF (PL-F-P) increases vascular density. (A) Smooth muscle cells were stained with an antibody against smooth muscle actin (SMA) and arteriole numbers were counted (magnification, ×200). Six fields were randomly selected from the infarct border zone of each myocardial section. (B) Endothelial cells were stained with an antibody against von Willebrand factor (vWF) and capillary numbers were counted (magnification, ×400). Six fields were randomly selected from the infarct border zone of each myocardial section. (C) Quantitative analysis of SMA^+^ vessels. ^**^P<0.01 versus the control group; ^△^P<0.05 versus the PL-F-P group. (D) Quantitative analysis of vWF+ vessels. ^**^P<0.01 versus the control group; ^△△^P<0.01 versus the PL-F-P group. bFGF, basic fibroblast growth factor; PDGF, platelet-derived growth factor; PL-bFGF, plasmid encoding bFGF; PL-PDGF, plasmid encoding PDGF.

Immunostaining of the infarcted myocardium for the expression of vWF and SMA demonstrated the augmentation of neovascularization in all the therapy groups (Fig. 3). Among the three therapy groups, PL-F-P showed the most marked vessel growth-stimulating effect. The arteriole density (SMA-positive rate) in the PL-F-P group increased by 23.9% (P<0.05) compared with that in the PL-bFGF group and 37.8% compared with that in the PL-PDGF group (P>0.05). However, for the arterioles, the difference between the PL-F-P and PL-PDGF groups was not statistically significant (Fig. 3C). The respective increases in capillary density (vWF-positive rate) were 61.5 and 167% (both P<0.01) compared with that of PL-bFGF and PL-PDGF groups (Fig. 3D).

## Discussion

Since therapeutic angiogenesis was suggested by Höckel *et al* (14) in 1993, studies on therapeutic angiogenesis have been performed globally. A number of growth factors, including FGF-1, FGF-2, FGF-4, vascular endothelial growth factor-121 (VEGF121), VEGF165 and VEGF-2, have been shown to exert angiogenic effects in preclinical and clinical studies (15–19). A small, double-blind, randomized trial suggested the clinical efficacy of sustained-release FGF-2 implanted in the myocardium during surgery. In addition, a phase I trial indicated that recombinant human VEGF improved myocardial perfusion at rest, possibly via a dose-dependent effect (20). However, to date, all published results from large clinical studies, including the FGF Initiating RevaScularization Trial (FIRST) (21), the Vascular endothelial growth factor in Ischemia for Vascular Angiogenesis (VIVA) trial (22) and the Angiogenic GENe Therapy-3 and 4 (AGENT-3 and -4) trials (15), have revealed no or modest therapeutic effects, with no agent or delivery strategy yet to demonstrate success in phase III testing. The disappointing results of these large clinical trials are reflective of the slow progress in the field of therapeutic angiogenesis. One of the factors contributing to the failure of large clinical trials may be that all the trials to date have been based on a treatment strategy using a single growth factor. Given the biological complexities of collateral blood vessel formation, more sophisticated strategies using a combination of growth factors may be required.

The data from the present study indicate that compared with single gene therapy, plasmid-mediated dual gene transfer of bFGF and PDGF resulted in a large increase in the number of capillaries and arterioles in ischemic muscle 28 days subsequent to infarction. In addition, compared with single gene-treated groups, the dual gene treatment group showed much larger arterioles, with increased pericyte coverage. These results suggest a synergistic effect between bFGF and PDGF during the process of neovascularization. A possible mechanism for this synergistic effect may be that bFGF acts to stimulate a robust angiogenic response and also upregulates PDGF receptor (R)-α and PDGF R-β expression. In this manner, PDGF-BB (PDGF homodimer), an active ligand for the PDGF-α and PDGF-β receptors, may subsequently display increased and potent angiogenic activity (9).

Compared with viral methods, the delivery of plasmid DNA is limited by lower gene transfer efficiency rates and a shorter half-life. However, skeletal muscle has been indicated to take up and express naked DNA more efficiently than other types of tissues (23). The present study showed that plasmid-mediated gene delivery is sufficient to achieve meaningful therapeutic effects. In addition, the results of the present study indicate that a short duration of exposure of ischemic tissue to PDGF and bFGF is sufficient to establish stable and functional blood vessels. This observation may lead to a change in the current therapeutic strategy, based on sustained exposure to angiogenic factors, to the ‘one-shot’ delivery of PDGF and bFGF. The mechanism underlying these observations may be that PDGF receptors aggregate on the cell surface, and although the initial aggregates are triggered by PDGF, the remainder may become autophosphorylated following the removal of PDGF. Such a mechanism for PDGF receptor activation in the absence of PDGF has been described previously (24). Thus, constant activation of PDGF receptors on endothelial and mural cells may lead to the increased stability of newly formed vessels, even following the removal of genous PDGF and bFGF (9). The additional advantage of plasmid-mediated gene transfer is that immune and inflammatory responses induced following the delivery of viral vectors are not stimulated following the transfer of naked plasmid DNA (25).

In the present study, the results of the echocardiography and examination of infarct size were consistent with those obtained from blood vessel measurements. Echocardiography showed a significant improvement in cardiac function in the dual gene-treated group. The examination of infarct size revealed that the average infarct size was significantly decreased in the dual gene-treated group. These results suggest that neovascularization was important for recovery following myocardial ischemia. The number of mature vessels determines the number of surviving myocardial cells; these cells are important for the improvement of cardiac function.

Recently, bFGF and PDGF have been shown to exert a synergistic effect that promotes the proliferation and migration of endothelial progenitor cells (EPCs) and increases VEGF release (26). The results of the present study were consistent with those of Cao *et al* (9), in which a synergistic effect was identified between bFGF and PDGF in a mouse cornea model and a rat/rabbit ischemic hind limb model (9). In addition, Hao *et al* (25) showed that the combination of bFGF and PDGF increased the number of capillaries and arterioles in the rat myocardial infarction model (25). In the present study, instead of using repeated injections of protein and multiple plasmids, the growth factors were delivered using an intramyocardial injection of single plasmid DNA with two transcription units, allowing the combined expression of two genes of interest. A gene delivery method was selected for a number of reasons, as follows: i) Using a gene delivery method targets the desired cells or tissues and minimizes signal propagation to non-target cells and tissues; ii) the gene delivery method avoids the side-effects caused by the systematic administration of recombinant proteins and viral carriers (27); iii) the expression of two genes of interest under a single promoter usually leads to a lower expression efficiency of the second gene. Furthermore, it is hard to control the expression efficiency and quantity of two genes cloned in two separate vectors. However, a multigenic cloning plasmid with two transcription units may be used to achieve controllable and independent gene expression *in vivo*.

Single plasmid-mediated dual gene therapy with bFGF and PDGF was effective in stimulating the functional and morphological maturation of the vasculature in cardiac muscle following infarction. The results of the present study may be relevant to future clinical trials involving angiogenic factors for the treatment of myocardial ischemic disease.

## Figures and Tables

**Table I:** Cardiac function 28 days subsequent to infarction.

Group	LVDd (mm)	LVDs (mm)	FS (%)	LVEF
Control	5.83±0.02	3.34±0.01	44.54±0.11	0.82±0.01
PL-bFGF	5.81±0.02^a^,^c^	2.56±0.02^b^,^c^	56.78±0.13^b^,^c^	0.91±0.01^b^,^c^
PL-PDGF	5.52±0.02^b^,^c^	2.56±0.02^b^,^c^	54.50±0.16^b^,^c^	0.92±0.01^b^,^c^
PL-F-P	5.41±0.01^b^	1.82±0.02^b^	65.52±0.13^b^	0.96±0.01^b^

a P<0.05 and

b P<0.01 versus the control group;

c P<0.01 versus the PL-F-P group.

LVDd, left ventricular end-diastolic diameter; LVDs, left ventricular end-systolic diameter; FS, fractional shortening; LVEF, left ventricular ejection fraction; PL-bFGF, plasmid encoding basic fibroblast growth factor; PL-PDGF, plasmid encoding platelet-derived growth factor; PL-F-P, plasmid encoding bFGF and PDGF.
